# Complementary multi‐omics profiling of chronic thromboembolic pulmonary hypertension reveals immune cell alterations, epigenetic changes, and genetically supported candidate genes

**DOI:** 10.1002/ame2.70166

**Published:** 2026-03-31

**Authors:** Xiaopeng Liu, Xia Zheng, Yajun Zhang, Yinghui Fang, Yanan Zhen

**Affiliations:** ^1^ Department of Cardiovascular Surgery China‐Japan Friendship Hospital Beijing China; ^2^ Department of Anesthesiology China‐Japan Friendship Hospital Beijing China

**Keywords:** biochemical indicators, chronic thromboembolic pulmonary hypertension, Mendelian randomization, multi‐omics integration

## Abstract

**Background:**

Chronic thromboembolic pulmonary hypertension (CTEPH) is driven by unresolved pulmonary arterial thrombi and involves complex processes such as vascular remodeling and immune dysregulation. Early identification of molecular markers may support more accurate diagnosis and individualized therapy.

**Methods:**

We integrated anthropometric and biochemical data with single‐cell RNA sequencing (scRNA‐seq), DNA methylation, and Mendelian randomization (MR) analyses. scRNA‐seq data were analyzed to determine altered cell populations and pathways. MR and colocalization analyses were conducted to identify genetically supported candidates related to CTEPH.

**Results:**

scRNA‐seq analysis revealed altered immune composition, with a modest increase in NK cells and an angiogenesis‐associated enrichment of monocytes and HSC‐G‐CSF cells in CTEPH patients, accompanied by activation of toll‐like receptor and MAPK signaling pathways. MR and colocalization identified several genetically associated genes—CLEC7A, TNFSF13B, LRP1, ETS1, and FGR—of which ETS1 and FGR demonstrated good diagnostic performance. DNA methylation analysis indicated marked alterations in chromatin assembly and epigenetic regulation.

**Conclusions:**

This multi‐omics study highlights critical genes, epigenetic features, and immune‐related cell populations associated with CTEPH. These findings improve understanding of disease mechanisms and offer potential biomarkers for early diagnosis and personalized management.

## Graphical abstract

This study presents an integrative multi‐omics framework to uncover the molecular mechanisms and potential biomarkers of chronic thromboembolic pulmonary hypertension (CTEPH). Anthropometric and biochemical data were correlated using canonical correlation analysis, revealing key cardiometabolic associations. Single‐cell RNA sequencing identified immune and stem cell subpopulations—particularly monocytes and HSC–G‐CSF cells—enriched in angiogenesis‐ and inflammation‐related pathways. Bulk RNA‐seq deconvolution with the BayesPrism algorithm quantified cell‐type composition, whereas Mendelian randomization and colocalization analyses identified causally linked hub genes (CLEC7A, TNFSF13B, FGR, LRP1, ETS1) with diagnostic potential. DNA methylation profiling further highlighted epigenetic dysregulation in vascular remodeling. Together, these integrated multi‐omics analyses delineate the molecular landscape of CTEPH and propose novel biomarkers and therapeutic targets for precision medicine.

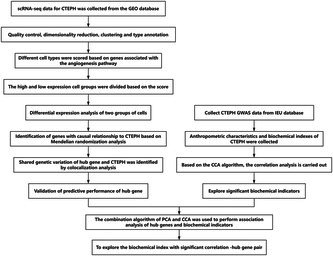

## 1 INTRODUCTION

Chronic thromboembolic pulmonary hypertension (CTEPH) is a form of pulmonary arterial hypertension (PAH), primarily caused by unresolved thrombi in the pulmonary arteries, ultimately leading to right heart failure.^1^, ^2^, ^3^ Its pathogenesis is intricate, typically involving processes such as hemostasis and vascular remodeling. Clinically, CTEPH presents nonspecifically, including symptoms like dyspnea, fatigue, and chest pain.^4^ Treatment options for CTEPH include interventional therapies, surgery, and anticoagulant treatments. In recent years, targeted therapies such as endothelin receptor antagonists^5^ and phosphodiesterase‐5 inhibitors^6^ have become mainstream treatments. The efficacy of these treatments is closely linked to the molecular target characteristics of patients, making individualized therapeutic strategies increasingly important. Radiomics feature identification provides new perspectives for early diagnosis and evaluation of therapeutic efficacy in CTEPH. High‐resolution imaging technology can be used to analyze changes in pulmonary vascular morphology and hemodynamic characteristics. Combined with clinical feature analysis, this aids in enhancing diagnostic accuracy, optimizing treatment plans, and predicting prognosis, thus contributing to a better understanding of the pathophysiological process of CTEPH and advancing precision medicine.

Multiple biochemical indicators obtained through current clinical testing can provide clues regarding the diagnosis and severity of CTEH. D‐dimer levels serve as an essential biochemical marker as they reflect ongoing coagulation and fibrinolysis within the body; elevated D‐dimer levels often indicate persistent thrombus formation.^7^ Additionally, troponin concentration testing is critical as it can indicate myocardial damage; increased right ventricular pressure due to CTEPH may lead to myocardial injury and elevate troponin levels.^8^ Another noteworthy indicator is N‐terminal pro‐brain natriuretic peptide (NT‐proBNP), whose elevated concentration is usually associated with increased cardiac load, particularly in cases of right heart dysfunction; changes in this indicator can help assess the severity of the disease and the effectiveness of treatment.^9^

On the contrary, genetic factors play a significant role in the susceptibility to CTEPH. Genomic techniques such as genome‐wide association studies (GWAS) can identify susceptibility genes associated with CTEPH. Single‐cell RNA sequencing (scRNA‐seq) technology enables the analysis of different cell types and their gene expression changes at the single‐cell level within lung tissue. For example, Lin et al. found that the upregulation of ARRDC3 accompanied by infiltration of various immune cells is involved in the development of CTEPH.^10^ Wang et al. discovered that TNF‐α promotes VSMC proliferation and migration by increasing FOS expression, driving the pathogenesis of CTEPH.^11^ Zhang et al. identified five hub genes associated with oxidative stress in CTEPH through comprehensive bioinformatics analysis.^12^ However, these studies perform single‐omics analyses based on RNA‐seq data alone, unable to comprehensively decipher the molecular characteristics of CTEPH from a multi‐omics perspective.

By integrating clinical features with multi‐omics genetic characteristics and exploring their correlations, it will contribute to a deeper understanding of the pathogenic mechanism of CTEPH and provide new prognostic assessment tools and therapeutic strategies for clinical practice. Furthermore, the pathogenesis of CTEPH involves vascular remodeling of the pulmonary arteries^13^ and neovascularization,^14^ which is mainly regulated by growth factors such as vascular endothelial growth factor (VEGF).^15^ Specifically, this paper first collected matched anthropometric and clinical data from real patients and selected top features with significant associations through correlation analysis. Further analysis of the expression landscape of angiogenesis‐related genes in the scRNA‐seq and RNA‐seq of CTEPH, combined with the analysis of DNA methylation data and Mendelian randomization (MR) analysis, provided a comprehensive genetic landscape.

CTEPH is caused by persistent thromboembolic obstruction of the pulmonary arteries, leading to pulmonary vascular remodeling, elevated pulmonary vascular resistance, and right heart failure.^1^, ^2^, ^3^ Because symptoms such as dyspnea and fatigue are nonspecific,^4^ early diagnosis and risk stratification remain challenging. Current treatments (pulmonary endarterectomy, balloon pulmonary angioplasty, targeted drugs, and anticoagulation) improve outcomes in many patients,^5^, ^6^ but therapeutic response is heterogeneous, implying unrecognized molecular subtypes. Routine biochemical markers—including D‐dimer, troponin, and N‐terminal pro–brain natriuretic peptide (NT‐proBNP)—reflect thrombotic activity and right ventricular strain,^7^, ^8^, ^9^ yet they offer limited insight into the cellular and molecular pathways that drive disease progression.

Recent transcriptomic studies have implicated immune cell infiltration, vascular smooth muscle cell activation, and oxidative stress–related hub genes in CTEPH,^10^, ^11^, ^12^ but most analyses are single‐omics and cannot link clinical phenotypes to cell‐type–specific programs, epigenetic changes, and genetic susceptibility. Given that CTEPH involves profound pulmonary vascular remodeling and neovascularization regulated by angiogenic pathways such as VEGF signaling,^13^, ^14^, ^15^ a multilayer view focused on angiogenesis and immune regulation may better capture key disease mechanisms.

Therefore, we adopted a complementary multi‐omics approach that sequentially integrates (i) anthropometric and biochemical data from CTEPH patients and controls, (ii) scRNA‐seq profiling of immune cell populations and angiogenesis‐related gene expression, (iii) DNA methylation analysis of whole‐blood samples, and (iv) Mendelian randomization and colocalization based on external GWAS and eQTL datasets. Our aim is to connect clinical features with cellular, epigenetic, and genetic information, thereby identifying candidate cell populations and genes associated with CTEPH and generating hypotheses for future mechanistic and translational studies.

## 2 METHODS

### 2.1 Data acquisition

For clinical data, we collected information from 25 patients diagnosed with CTEPH within our institution; detailed information is provided in Table 1. Additionally, this study utilized scRNA‐seq data from the Gene Expression Omnibus (GEO) database's GSE274381 dataset, which included five CTEPH samples (GSM8448139, GSM8448140, GSM8448141, GSM8448142, GSM8448143) and two control samples (GSM8448144 and GSM3169075). RNA‐seq data were collected from 14 CTEPH and 4 control samples in the GSE130391 dataset. DNA methylation array data were obtained from the GSE113061 dataset, which included five CTEPH and three control samples. For Mendelian randomization (MR) analysis, GWAS summary statistics from the “finn‐b‐I9_HYPTENSPUL” dataset were collected from the FinnGen database (https://www.finngen.fi/en/access_results), containing 477 PAH cases and 372 077 controls. Summary statistics for expression quantitative trait loci (eQTLs) of specified genes were collected from the IEU database (https://gwas.mrcieu.ac.uk/).

**TABLE 1:** Clinical information of the samples.

Characteristics	Female (*n* = 17)	Male (*n* = 8)	*p* Value
Age (year, mean ± SD)	46.059 ± 11.02	57.5 ± 8.9762	0.018
Height (cm, mean ± SD)	175.59 ± 5.304	161.25 ± 7.0862	<0.001
Weight (kg, mean ± SD)	78.412 ± 11.479	63.625 ± 12.66	0.008
BMI (mean ± SD)	25.371 ± 3.0865	24.669 ± 5.6636	0.689
Body surface area (m^2^, mean ± SD)	1.9341 ± 0.16568	1.6525 ± 0.15827	<0.001
Smoking history (1 = ever/current, 0 = never)	0 (0%)	8 (32%)	0.026
History of hypertension, *n* (%)	3 (12%)	1 (4%)	0.081
History of diabetes, *n* (%)	0 (0%)	1 (4%)	1.000
History of coronary heart disease, *n* (%)	1 (4%)	2 (8%)	1.000

Abbreviations: BMI, body mass index; SD, standard deviation.

### 2.2 Correlation analysis of anthropometric features and biochemical markers

This study utilized canonical correlation analysis (CCA) to evaluate the correlation between anthropometric features and biochemical markers in CTEPH. CCA maximizes the correlation between two different modalities' linear combinations to find significant features during the correlation analysis process. Let the anthropometric feature matrix and biochemical marker matrix be $X\in R^{n\times p}$ and $Y\in R^{n\times q}$, respectively, where *n*, *p*, and *q* represent the number of samples, anthropometric features, and biochemical markers, respectively. Below is the objective function of the algorithm:

$$\mathrm{maximize}\frac{\mathrm{Cov}\left(U,V\right)}{\sqrt{\mathrm{Var}\left(U\right)\cdot \mathrm{Var}\left(V\right)}}$$

Here, U and V are two typical variables generated by linear combinations, representing the linear combinations of anthropometric features and biochemical markers, respectively. $\mathrm{Cov}\left(U,V\right)$ represents the covariance of U and V, and $\mathrm{Var}\left(U\right)$ and $\mathrm{Var}\left(V\right)$ are the variances of U and V,respectively.a and b are two weight vectors, used to weigh the variables in *X* and *Y*, forming the typical variables U and V:

$$U=a^{T}X$$

$$V=b^{T}Y$$

Solving the generalized eigenvalue problem yields a and b, maximizing the corresponding correlation. This study implemented CCA algorithm solving via Matlab (version R2021b). To further assess the statistical significance of the relationships between the original variables and their corresponding canonical variates, Pearson correlation coefficients and associated *p*‐values were computed. Each *p*‐value quantifies the likelihood that the observed correlation arises by chance, thereby providing a statistical measure of the importance of each variable's contribution to the canonical variate.

### 2.3 Analysis methods for scRNA‐seq and RNA‐seq data

For scRNA‐seq data, this study performed data reading, quality control, normalization, identification of highly variable genes, dimensionality reduction, clustering, and visualization of cell types and distributions using the “Seurat” package (version 4.3.0). During quality control, cells were retained only if they expressed between 200 and 6000 genes, had a total UMI count ≤50 000, a mitochondrial gene proportion ≤20%, and a ribosomal gene ratio >1%. Cells that did not meet these thresholds were filtered out prior to downstream analysis. Data were normalized using Seurat's LogNormalize function. Highly variable genes were selected via FindVariableFeatures(), followed by prinicipal component analysis (PCA)‐based dimensionality reduction. Batch effect correction across the six samples was achieved using the “Harmony” package. Clustering was performed using the Louvain algorithm with a resolution of 0.8, and we used the singleR package and the classic marker genes of various cell types to determine the annotation of cell types based on the reference transcriptome data. Cell‐type annotation was performed with the “singleR” package. Visualization of cellular composition was carried out using the “ggplot2” package. Additionally, we collected hallmark gene sets involved in angiogenesis pathways (specific gene lists are available in the supplementary material file “HALLMARK_ADIPOGENESIS.csv”). Five scoring methods (AUCell, UCell, singscore, ssGSEA, and Addmodulescore(·)) were used to explore the scores of this gene set across different cell types. Based on the median value of cellular scores, all cells were divided into high‐ and low‐expression groups. Gene set variation analysis (GSVA) was performed on the two cell groups using the “GSVA” package, and pathway significance was determined based on Benjamini–Hochberg FDR–adjusted *p*‐values (<0.05). Differentially expressed genes (DEGs) between the two groups of cells were identified using the FindAllMarkers(·) function in the Seurat package, with statistical significance assessed by the Wilcoxon rank‐sum test and adjusted for multiple testing using the Benjamini–Hochberg false discovery rate (FDR) method (adjusted *p* < 0.05).

For the RNA‐seq dataset, data were downloaded and organized using the “GEOquery” package. The GSE274381 dataset was used as a reference for cell‐type‐specific expression profiles to deconvolute the GSE130391 dataset. Ribosomal, sex chromosome, and mitochondrial genes were excluded. The GSE130391 dataset was used to evaluate the infiltration abundance of each cell type, grouping by cell type and high/low‐expression cell clusters separately.

To analyze the GSE130391 dataset, the BayesPrism algorithm implemented in the R package “BayesPrism” was applied, using the GSE274381 dataset as a reference for cell‐type‐specific expression profiles. Prior to deconvolution, single‐cell count matrices and corresponding cell‐type annotations were preprocessed to generate BayesPrism‐compatible input files. To minimize noise and bias, genes with high expression but low cell‐type specificity were filtered out using the cleanup.genes() function. Additionally, cell‐type correlation heatmaps and outlier detection plots were generated to evaluate data quality and intercellular relationships. Figure S1 presents the overall process of data collection for this study.

### 2.4 Analysis method for DNA methylation data

In this paper, DNA methylation array data from five CTEPH and three control whole blood samples were subjected to quality control and differential analysis using the “RnBeads” package^16^ in R software. Ranking scores were calculated based on the average difference between groups, the relative effect size (log2 of the methylation quotient), and the area under the precision–recall curve (AP). Because the methylation data were generated from heterogeneous whole blood rather than purified cell populations, we did not perform explicit reference‐based cell‐type deconvolution or composition adjustment (e.g., Houseman correction), and the resulting differential methylation signals may therefore reflect a combination of cell‐intrinsic epigenetic changes and shifts in leukocyte composition.

### 2.5 MR and colocalization analysis

GWAS summary data for PAH were screened through the FinnGen database, extracting genetically supported candidates associated with eQTL summary data related to DEGs. Single‐nucleotide polymorphism (SNPs) associated with *p* < 1e−8 for each gene were selected as potential instrumental variables (IVs), and linkage disequilibrium (LD) among SNPs was calculated. SNPs with *R* ^2^ < 0.001 (clumping window size = 10 000 kb) were retained if *F* > 10. Four statistical methods (IVW, MR Egger, weighted median, weighted mode) were employed; if there was only one SNP, the Wald ratio method was used. Finally, the genetically supported candidates were verified through heterogeneity tests (such as Cochran's IVW *Q*‐test) and analyzed using the R package “TwoSampleMR.” A leave‐one‐out approach was adopted to evaluate the impact of specific genetic variations on outcome risk by recalculating the combined effect size after excluding each SNP, identifying and removing variants with excessive influence to assess the robustness of the results. Because large CTEPH‐specific GWAS summary statistics suitable for well‐powered two‐sample MR are currently limited, we used a FinnGen PAH GWAS as a pulmonary‐hypertension–related proxy phenotype to prioritize genetically supported candidate genes relevant to shared PH mechanisms (pulmonary vascular remodeling and pressure overload), and interpreted MR results as indirect.

Summary‐data‐based Mendelian randomization (SMR) analysis was conducted using SMR software (version 1.3.1).^17^ The final threshold was determined with a threshold of *p* < 0.05 and HEIDI *p* > 0.05. This analysis provided further support for genetically supported candidates for PAH. Lastly, colocalization analysis of eQTL and PAH GWAS was performed using the R package “coloc,” calculating the posterior probability within a 500 kb region around index SNPs, with SNP.PP.H4 >0.95 serving as the colocalization threshold.

### 2.6 Statistical analysis

Bioinformatics and statistical analyses were conducted using R software (version 4.1.2). Correlation was assessed based on Spearman's correlation coefficient. Differences in gene expression/methylation levels between the two groups were evaluated using the Wilcoxon test.

## 3 RESULTS

### 3.1 Results of the correlation analysis between anthropometric characteristics and biochemical indicators

**FIGURE 1:**
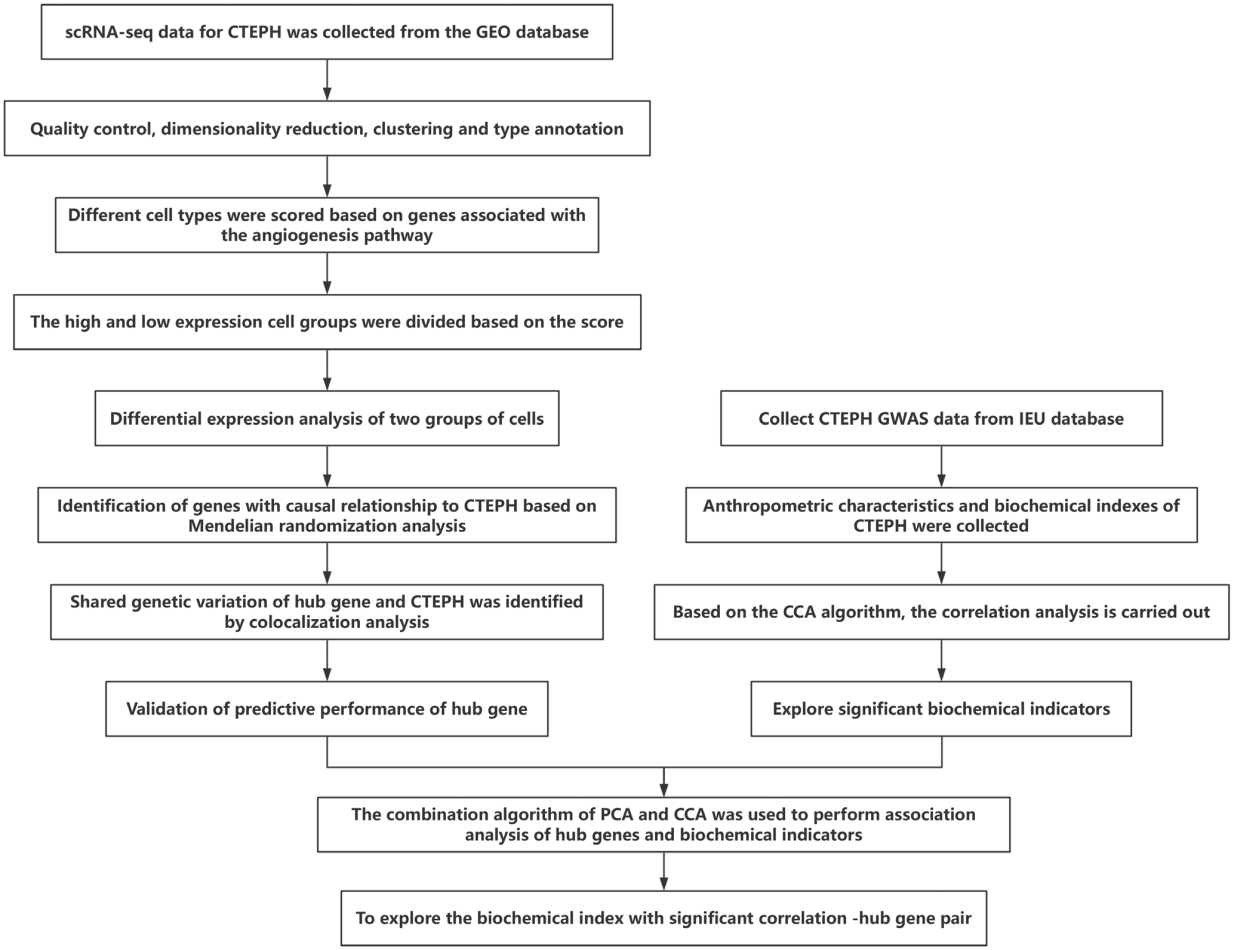
Technical roadmap for the paper.

Figure 1 provides an overview of the technical roadmap of this study. Initially, the CCA algorithm was applied to integrate anthropometric characteristics and biochemical indicators of 25 CTEPH patients (Figure S2A,B). Furthermore, in Section 1.1 and Figure S2 of the supplementary materials, the data information of this paper is summarized. It can be observed that there is a significant correlation between anthropometric characteristics and biochemical indicators in CTEPH patients. On the left side, the canonical variable weights of anthropometric characteristics (age, height, weight, and BMI [body mass index]) are displayed, with weight having the highest weight value (0.74), followed by height (−‐0.58); BMI and age have relatively smaller weights at −0.34 and −0.058, respectively. On the right side, the weights of the biochemical indicators are shown, with hemoglobin (Hemoglobin) having the highest weight with the first canonical variable, at 0.39, whereas NT‐proBNP has a weight of −0.49, indicating a strong negative correlation with the biochemical indicators. These results indicate that in CTEPH patients, there is a significant correlation between indicators such as weight and NT‐proBNP and other variables. Further analysis of the correlation between biochemical indicators and anthropometric characteristics is presented. Among the biochemical indicators, hemoglobin (Hemoglobin), NT‐proBNP, low‐density lipoprotein (LDL), and troponin show higher weights. Notably, the negative weight of NT‐proBNP (−0.49) is significant, which relates to cardiac dysfunction in CTEPH patients. On the contrary, hemoglobin (Hemoglobin) has a strong positive weight (0.39), suggesting its potential significance in the hematological characteristics of CTEPH patients. The correlation analysis (Figure S2C) reveals a strong correlation between LDL and troponin, further illustrating the potential value of these biochemical indicators in assessing the condition of CTEPH patients. As the variable with the highest weight (0.74) among anthropometric characteristics, the high correlation between weight and biochemical indicators also reflects the importance of weight in the pathological process of CTEPH patients.

### 3.2 Expression landscape of angiogenesis pathway‐related genes in CTEPH scRNA‐seq data

Using the Seurat software package, we performed read alignment, quality control (QC), normalization, selection of highly variable genes (Figure S3A), dimensionality reduction, batch correction (Figure S3B), and clustering on the scRNA‐seq data of both CTEPH and control groups. Harmony integration reduced the batch‐explained variance from *R* ^2^ up to 0.73 in PCA components to <0.03 across all Harmony dimensions, confirming successful and effective batch correction (Tables S1 and S2). Specifically, highly variable genes like IGHG1, MZB1, and IGLC2 were identified as key features promoting cell clustering. After batch correction using Harmony, T‐distributed Stochastic Neighbor Embedding (t‐SNE) and Uniform Manifold Approximation and Projection (UMAP) revealed distinct cell clusters including T cells, monocytes, NK cells, B cells, and hematopoietic stem cells (HSCs) (Figure S3C,D). In the global cell‐type composition analysis, the overall proportion of NK cells was moderately higher in CTEPH samples than in healthy controls (Figure S3F), indicating a shift in the NK‐cell compartment.

**FIGURE 2:**
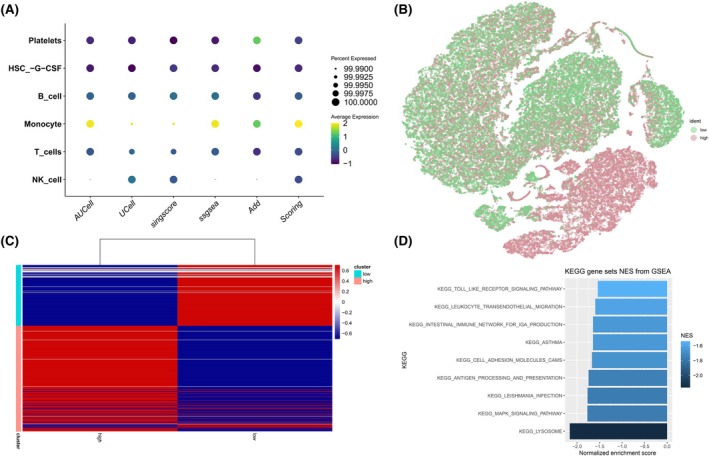
Angiogenesis pathway–based stratification of immune cell states in chronic thromboembolic pulmonary hypertension (CTEPH) single‐cell RNA sequencing (scRNA‐seq) data. (A) Bubble plot summarizing angiogenesis pathway activity across major immune cell types. For each cell type, enrichment scores were computed using five single‐cell gene set scoring methods (AUCell, UCell, singscore, ssGSEA, and AddModuleScore) applied to angiogenesis‐related gene sets. Bubble color represents the mean standardized angiogenesis score (dimensionless) across methods, and bubble size indicates the proportion of cells within each cell type with a positive angiogenesis score. (B, C) Two‐dimensional Stochastic Neighbor Embedding (t‐SNE) (B) and Uniform Manifold Approximation and Projection (UMAP) (C) embeddings of all cells, colored by angiogenesis program activity. Cells were dichotomized into angiogenesis high and angiogenesis low clusters using the median of an integrated angiogenesis score. The *x*‐ and *y*‐axes correspond to t‐SNE/UMAP embedding dimensions without physical units. (D) Bar plot of GSEA results comparing angiogenesis‐high versus angiogenesis‐low cell clusters. Bars display the normalized enrichment score (NES) for significantly enriched pathways; bar length is proportional to NES, and color indicates pathways upregulated in angiogenesis‐high (red) or angiogenesis‐low (blue) clusters. Corresponding false discovery rate (FDR)‐adjusted *q*‐values and full pathway lists are provided in the Supplementary Materials.

Based on the analysis of scRNA‐seq data, we further investigated the differences in cellular composition and functional aspects between CTEPH patients and healthy controls. Based on the angiogenesis pathway gene sets, we next scored all cells using multiple methods (AUCell, UCell, singscore, ssGSEA, and AddModuleScore) and divided them into high‐ and low‐score groups (Figure 2A). Within this angiogenesis‐high compartment, monocytes and HSC‐G‐CSF cells were preferentially enriched in CTEPH samples, suggesting that these myeloid populations predominantly carry the angiogenesis‐related transcriptional program. Figure 2B,C visualizes the cellular distribution of high‐ and low‐expressing populations via t‐SNE and UMAP, indicating that certain cell populations are distinctly distributed in CTEPH patients. Subsequent Gene Set Enrichment Analysis (GSEA) identified multiple significantly enriched pathways in the high‐expression cluster, including toll‐like receptor signaling, leukocyte transendothelial migration, MAPK signaling, and cell adhesion molecules (CAMs) (Figure 2D).

### 3.3 Results of MR and colocalization analysis of DEGs

In this section, we identify genetically associated genes of PAH from DEGs through two‐sample MR analysis. Specifically, we collected eQTL summary statistics for DEGs from the IEU database and PAH GWAS summary statistics from the Finnish database. Using the “TwoSampleMR” package in R software, we identified genetically supported candidates for PAH. The outcome ID finngen_R9_J10_PULMOEDEMA was derived from GWAS summary data involving 372 554 samples (cases: 477; controls: 372 077). After reading the exposure and outcome data, SNPs with *p* < 5e−8 were selected as working instruments, and SNPs with *F* > 10 and *R* ^2^ < 0.001 were chosen by calculating linkage disequilibrium between SNPs. In the end, seven genes corresponding to eQTL data showed positive relationships (Figure S4A–G). The results indicated that CLEC7A (IVW method; *p* = 0.044; 95% confidence interval [CI]: 1.0062–1.6031), COTL1 (IVW method; *p* = 0.012; 95% CI: 0.1879–0.8183), ETS1 (WR method; *p* = 0.029; 95% CI: 0.0709–0.8725), FGR (IVW method; *p* = 0.0007; 95% CI: 0.2817–0.7131), LRP1 (IVW method; *p* = 0.027; 95% CI: 1.1233–7.6199), PILRA (IVW method; *p* = 0.043; 95% CI: 0.2704–0.9822), and TNFSF13B (IVW method; *p* = 0.028; 95% CI: 1.0437–2.1751) had relationships with PAH (Figure S4H). Increased expression of FGR, PILRA, COTL1, SAT1, and ETS1 had a relationship with lower risk of PAH, whereas increased expression of TNFSF13B, LRP1, and CLEC7A had a relationship with higher risk of PAH. The genes with positive relationships were designated as hub genes for subsequent analysis. Tables S3 and S4, respectively, present the results of the Heterogeneity and Pleiotropy analyses for the hub genes.

**FIGURE 3:**
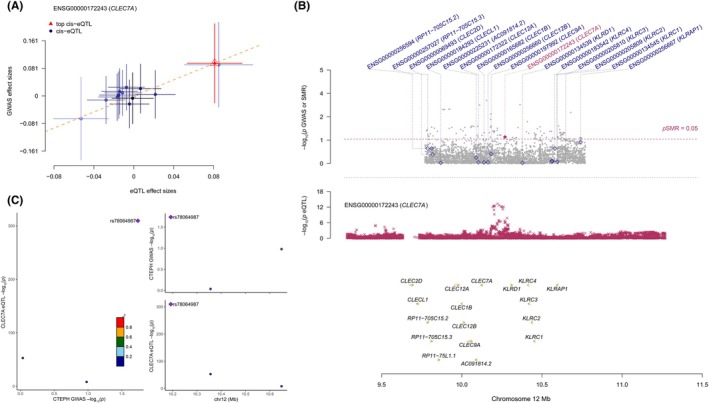
Mendelian randomization and colocalization analysis of CLEC7A with pulmonary arterial hypertension (PAH). (A) Summary‐data‐based Mendelian randomization (SMR) effect plot for CLEC7A. Each point represents a genetic variant used as an instrumental variable. The *x*‐axis shows chromosomal position within the CLEC7A locus, and the *y*‐axis shows the SMR effect estimate (*β*) of CLEC7A expression on PAH risk, with the horizontal line indicating the genome‐wide significance threshold for the SMR test. Variants highlighted in red pass the SMR significance threshold. (B) Regional association (locus) plot for the CLEC7A region, integrating PAH GWAS and CLEC7A cis‐eQTL signals. The *x*‐axis denotes genomic position, whereas the left *y*‐axis shows –log10(*p*) values for PAH GWAS association, and the right *y*‐axis shows –log10(*p*) values for CLEC7A eQTL association. The lead variant used in the SMR analysis is indicated, and neighboring SNPs are colored according to their linkage disequilibrium with the lead SNP. (C) Colocalization analysis for CLEC7A expression and PAH risk at the same locus. Bars display the posterior probabilities for the five standard hypotheses (H0–H4), with H4 representing a shared causal variant influencing both CLEC7A expression and PAH susceptibility. A higher posterior probability for H4 indicates stronger evidence that the eQTL and GWAS signals arise from a common underlying causal variant.

Furthermore, to further eliminate the effects of pleiotropy and linkage disequilibrium, we conducted SMR and HEIDI tests to validate the MR analysis results. The results showed that CLEC7A was consistent with the MR results in terms of effect direction and passed the HEIDI test (*p* > 0.05) and the SMR test (*p* < 0.05) (refer to the “mysmr_blood.smr” file in the supplementary materials). The SMR site and effect plots are shown in Figure 3A,B. A colocalization analysis was performed at the eQTL‐GWAS level for this positive relationship, and the SNP.PP.H4 of CLEC7A‐PAH was above 0.95 (Figure 3C).

### 3.4 Validation of prediction performance of hub genes and cells

**FIGURE 4:**
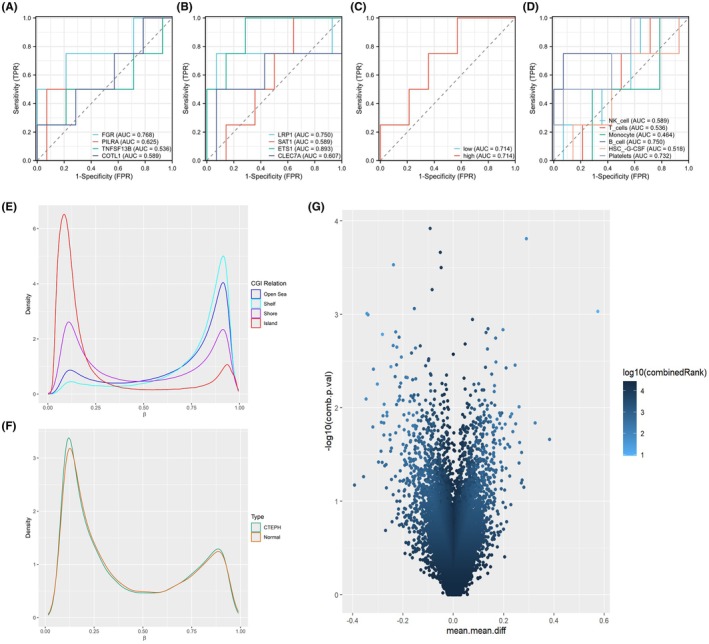
Transcriptomic and DNA methylation signatures associated with chronic thromboembolic pulmonary hypertension (CTEPH). (A, B) Receiver operating characteristic (ROC) curves based on bulk RNA‐seq data from CTEPH and control peripheral blood samples, evaluating the diagnostic performance of pulmonary arterial hypertension (PAH)‐related candidate genes. The *x*‐axis shows the false‐positive rate (specificity), and the *y*‐axis shows the true‐positive rate (sensitivity); curves are dimensionless, and the AUC is reported for each gene. (C, D) ROC curves derived from deconvoluted expression profiles obtained using the BayesPrism algorithm. Panel (C) displays ROC curves for high‐expression versus low‐expression cell groups defined by dichotomizing integrated gene expression scores, and panel (D) shows ROC curves for specific deconvoluted cell types. Axes represent false‐positive rate and true‐positive rate as in panels (A, B), with AUC values indicating classification performance in each cellular compartment. (E) Distribution of DNA methylation β‐values (ranging from 0 to 1) across different CpG island‐related regions (Island, Shore, Shelf, Open Sea) in CTEPH and control whole‐blood samples. Each density curve summarizes the genome‐wide β‐value distribution within the indicated region and group. (F) Overall methylation density curves comparing CTEPH and control groups, showing the global distribution of CpG β‐values across the array. (G) Volcano plot of Differentially Methylated Regions (DMRs) This work was supported by the National High Level Hospital Clinical Research Funding, China (Grant No. 2023‐NHLHCRF‐PY‐10) and the National High Level Hospital Clinical Research Funding – Elite Medical Professionals Project of China‐Japan Friendship Hospital, China (Grant No. ZRJY2023‐GG10). between CTEPH and controls. The *x*‐axis represents the difference in mean *β*‐value (Δ*β*) between groups, and the *y*‐axis shows –log10(adjusted *p*‐value). Red and blue points highlight regions with large Δ*β* and statistically significant differences according to the RnBeads differential methylation analysis, with full DMR lists provided in the Supplementary Materials.

We next evaluated the diagnostic performance of the MR‐prioritized hub genes using bulk RNA‐seq data from CTEPH patients and controls (Figure 4A,B). All eight genes achieved area under the curve (AUC) values above 0.5, including FGR (AUC = 0.766), PILRA (AUC = 0.625), TNFSF13B (AUC = 0.536), COTL1 (AUC = 0.589), LRP1 (AUC = 0.750), SAT1 (AUC = 0.689), ETS1 (AUC = 0.893), and CLEC7A (AUC = 0.607), with ETS1 demonstrating the highest discriminatory ability. To further assess cell‐type contributions, we applied the BayesPrism algorithm to deconvolute the CTEPH RNA‐seq samples using our scRNA‐seq dataset as reference. Receiver operating characteristic (ROC) analysis of the resulting infiltration estimates showed that the high‐ versus low‐expression cell groups yielded an AUC of 0.714. Among individual cell types, the AUC values were 0.589 for NK cells, 0.536 for T cells, 0.464 for monocytes, 0.750 for B cells, 0.518 for HSCs, and 0.732 for platelets. These findings indicate that the aggregated high/low‐expression groups, as well as B cells and platelets, exhibit relatively stronger predictive performance compared with other immune cell subsets (Figure 4C–D).

In the DNA methylation array data (five CTEPH vs. three controls), we observed clear differences in overall methylation patterns between groups. In particular, CpG sites located in Open Sea, Shelf, Shore, and Island regions showed distinct *β*‐value distributions, as illustrated by density and volcano plots (Figure 4E–G). To obtain preliminary functional insights, we performed Gene Ontology (GO) enrichment analysis on genes annotated from the top 100 hyper‐ and hypomethylated regions. At a descriptive level, hypermethylated regions were modestly enriched for terms related to chromatin organization, nucleosome assembly, and general epigenetic regulation of gene expression, whereas hypomethylated regions showed enrichment for pathways broadly linked to chromatin remodeling, angiogenesis‐related signaling, and immune processes. Given the very small sample size and the use of heterogeneous whole blood, these methylation and pathway findings should be regarded as exploratory and hypothesis‐generating rather than definitive evidence for specific developmental or immune mechanisms in CTEPH. Detailed GO term lists and pathway annotations have therefore been moved to the Supplementary Materials (“GO_pathway.xlsx”) for reference.

## 4 DISCUSSION

Although the study incorporates four molecular layers, the analyses were performed sequentially and not through a fully integrated multi‐omics model. Thus, the results should be interpreted as complementary evidence derived from independent omics modalities rather than as outcomes of a formal integrative framework. Future work applying joint multi‐omics factorization approaches will be valuable for constructing unified molecular signatures of CTEPH.

CCA was used to characterize multivariate relationships between biochemical indicators and anthropometric characteristics in CTEPH. This approach allows us to identify which clinical variables are most strongly linked to disease‐related biochemical profiles. In our analysis, weight showed the strongest loading among anthropometric traits (canonical weight = 0.74), suggesting a prominent role in the pathophysiological profile of CTEPH patients. Weight was positively associated with hemoglobin (0.39), LDL (0.28), and troponin (0.29), indicating that higher body weight may be linked to altered oxygen transport capacity, lipid metabolism, and increased cardiac stress. Notably, the correlation between weight and troponin—a marker of myocardial injury—supports the notion that excess weight can aggravate cardiovascular burden and impair cardiac function in CTEPH.^18^ Hemoglobin was positively correlated with red blood cell count and platelets, consistent with increased erythrocyte mass and blood viscosity potentially aggravating vascular resistance and right ventricular load in CTEPH.^19^ In contrast, NT‐proBNP showed negative correlations with several biochemical markers, particularly uric acid (−0.49), underscoring the interplay between cardiac dysfunction and metabolic abnormalities in this patient population.^9^, ^20^, ^21^ These observations align with the established role of NT‐proBNP as a heart failure biomarker and suggest that combined cardiac and metabolic impairment warrants close surveillance in CTEPH. Overall, these findings highlight the importance of weight management in CTEPH, as obesity and weight gain may exacerbate disease progression through mechanisms such as increased vascular resistance.^22^ In addition, biochemical markers including hemoglobin,^23^ NT‐proBNP, and uric acid^24^ show meaningful correlations in CTEPH and should be monitored closely to facilitate the early detection of cardiovascular and metabolic complications.

The scRNA‐seq analysis revealed notable alterations in the cellular landscape of CTEPH compared with healthy controls. At a global level, NK cells exhibited a modest expansion in CTEPH samples, whereas the angiogenesis‐high cell clusters were primarily composed of monocytes and HSC‐G‐CSF cells. The enrichment of monocytes in CTEPH is consistent with previous reports linking monocyte activation to the inflammatory responses and vascular remodeling that characterize the disease. Monocytes may contribute to a pro‐inflammatory milieu through cytokine secretion and interactions with endothelial cells, thereby promoting disease progression. To further characterize angiogenic signaling, all cells were scored using angiogenesis‐related gene sets and classified into high‐ and low‐expression groups based on the median score. GSVA analysis identified multiple pathways that differed significantly between these two groups, several of which have been previously implicated in CTEPH. For example, Xi et al. identified 679 tissue‐specific proteins in endarterectomized intimal tissues from CTEPH patients, enriched in pathways such as leukocyte transendothelial migration.^25^ In addition, Wu et al. demonstrated that tissue factor regulates autophagy in pulmonary arterial endothelial cells in CTEPH rats through the p38 MAPK‐FoxO1 pathway.^26^ These findings support the involvement of inflammatory and angiogenic signaling in CTEPH pathobiology and highlight relevant cellular populations contributing to these processes.

When assessing the clinical relevance of the identified biomarkers, it is important to interpret their diagnostic performance in a balanced way. Although several hub genes and immune cell types emerged as potential diagnostic markers for CTEPH, their discriminative ability varied considerably. Some genes, such as TNFSF13B (AUC = 0.536) and CLEC7A (AUC = 0.607), provided only modest predictive values, suggesting limited utility as stand‐alone diagnostic indicators. Likewise, certain immune cell subsets, including monocytes (AUC = 0.464) and T cells (AUC = 0.536), showed weak classification performance when evaluated individually. In contrast, ETS1 (AUC = 0.893) and LRP1 (AUC = 0.750) exhibited substantially higher diagnostic accuracy, indicating that they may represent more promising biomarker candidates. Overall, these results suggest that no single marker is sufficient for reliable diagnosis and that combining multiple molecular and cellular features in a multi‐parameter predictive model is likely to improve sensitivity and specificity. Integrating hub gene expression with established clinical parameters may yield a more comprehensive and clinically useful diagnostic framework for CTEPH. Future studies with larger, independent cohorts and formal model optimization will be needed to validate and refine these predictive signatures.

From the perspective of DNA methylation, the array data revealed global differences in CpG methylation patterns between CTEPH and controls, particularly in Open Sea and CpG island‐related regions. These observations are consistent with a potential contribution of epigenetic dysregulation to CTEPH; however, the very small sample size and the use of heterogeneous whole blood impose important limitations. Because we did not perform explicit cell‐type composition adjustment, the detected differential methylation signals may reflect a mixture of true cell‐intrinsic epigenetic changes and shifts in circulating leukocyte subsets. GO enrichment analysis of the most strongly hyper‐ and hypomethylated regions suggested the involvement of general chromatin organization, epigenetic regulation, and broad immune‐ and angiogenesis‐related pathways, but these results should be interpreted cautiously and regarded as hypothesis‐generating. We have therefore restricted the main‐text interpretation to these high‐level patterns and moved the more detailed pathway annotations to the Supplementary Materials.

Notably, for the planned clinical–molecular association analysis, the effective matched sample size was substantially smaller after harmonizing available biochemical and gene features (*n* = 8), and missing biochemical values were handled by mean imputation, which may further increase uncertainty in small‐sample settings. Robustness checks suggested statistical instability (in‐sample canonical correlation *r* = 1.0, permutation *p* = 0.786, and a degenerate bootstrap 95% CI of [1.0, 1.0, 1.0]), consistent with overfitting/degenerate solutions; therefore, we removed this association analysis from the final manuscript and report it here only as a limitation. Second, although CCA originally suggested associations between genes and biochemical indicators, these correlations do not establish causality and—given the instability noted above—should be interpreted with caution rather than as confirmatory evidence. The identified hub genes, such as CLEC7A, TNFSF13B, FGR, and ETS1, were inferred through bioinformatic and MR analyses but lack direct experimental or functional validation. Furthermore, no in vitro or in vivo experiments were performed in this study to verify the biological functions, regulatory effects, or mechanistic contributions of these genes, and this absence of experimental validation represents a key limitation of our current findings. The DNA methylation analysis was based on a very small number of whole blood samples (five CTEPH vs. three controls) without explicit correction for blood cell‐type composition, so the identified methylation differences and enriched pathways should be viewed as preliminary and may reflect both epigenetic changes and alterations in leukocyte subsets. Moreover, the lack of experimental evidence restricts the ability to confirm whether these genes truly drive the molecular and cellular alterations observed in CTEPH. Future research should incorporate in vitro and in vivo experiments—such as gene silencing, overexpression, or CRISPR‐based editing—to confirm the mechanistic roles of these genes in CTEPH pathogenesis. Moreover, integrating genomics, transcriptomics, metabolomics, and longitudinal clinical data will further enhance the understanding of the molecular mechanisms underlying CTEPH and support the development of targeted diagnostic and therapeutic strategies.

## 5 CONCLUSION

This study, through integrative multi‐omics analysis, has uncovered key molecular mechanisms and potential biomarkers of CTEPH. Based on CCA analysis, significant correlations between anthropometric characteristics and biochemical indicators in CTEPH patients were identified. The scRNA‐seq analysis indicated altered immune composition in CTEPH, characterized by a modest increase in NK cells and an angiogenesis‐associated enrichment of monocytes and HSC‐G‐CSF cells, highlighting the critical role of the immune system. MR analysis identified hub genes associated with the disease, such as CLEC7A, FGR, and TNFSF13B, and through methylation analysis, it revealed the importance of epigenetic regulation in CTEPH. These findings provide new targets for the diagnosis and personalized treatment of the disease.

## AUTHOR CONTRIBUTIONS

**Xiaopeng Liu:** Conceptualization; data curation; methodology; software; writing – original draft. **Xia Zheng:** Conceptualization; data curation; software; writing – original draft. **Yajun Zhang:** Conceptualization; software; writing – review and editing. **Yinghui Fang:** Conceptualization; data curation; methodology; writing – review and editing. **Yanan Zhen:** Conceptualization; methodology; supervision; writing – review and editing.

## FUNDING INFORMATION

This work was supported by the National High Level Hospital Clinical Research Funding, China (no.: 2023‐NHLHCRF‐PY‐10); the National High Level Hospital Clinical Research Funding‐Elite Medical Professionals Project of China‐Japan Friendship Hospital, China (no.: ZRJY2023‐GG10).

## CONFLICT OF INTEREST STATEMENT

The authors declare that they have no competing interests.

## ETHICS STATEMENT

This study was approved by the Ethics Committee of China‐Japan Friendship Hospital (approval number 2023‐KY‐246; approval date 2023/10/27). The clinical data analyzed were retrospective and anonymized, and the requirement for informed consent was waived by the committee.

## Supporting information


**Figure S1.** The data collection and analysis process of this paper.
**Figure S2.** Correlation analysis results of anthropometric characteristics and biochemical indexes. (A, B) anthropometric characteristics and biochemical index weight heat maps obtained by canonical correlation analysis (CCA) analysis. (C) The correlation analysis heatmap between the two.
**Figure S3.** The basic analysis results of single‐cell RNA sequencing (scRNA‐seq) data. (A) The volcano plot obtained by screening highly variable genes using the Seurat software package. (B) The distribution of scRNA‐seq data from chronic thromboembolic pulmonary hypertension (CTEPH) and control group samples in a two‐dimensional nonlinear space after batch correction. (C, D) The distribution of cell clusters in a two‐dimensional nonlinear space after different types of cell labeling, based on the t‐SNE and UMAP dimensionality reduction methods, respectively. (E) The expression of the marker gene in different types of cells. (F) Bar chart showing the distribution of different cell clusters across various samples.
**Figure S4.** The MR analysis results for differentially expressed genes (DEGs). Panels (A) through (G) show scatter plots of the MR analysis results for CLEC7A, COTL1, ETS1, FGR, LRP1, PILRA, and TNFSF13B with pulmonary arterial hypertension (PAH), respectively (the MR result for SAT1 was not plotted due to an insufficient number of SNP sites). (H) The MR analysis results for eight potential pathogenic genes. The horizontal dashed line corresponds to *p* = 3.59 × 10^−5^ (0.05/1394). “ln” stands for the natural logarithm; “OR” stands for odds ratio. “nSNP” represents the number of single‐nucleotide polymorphism (SNPs) used to estimate causal effects. “CI” stands for confidence interval.
**Table S1.** Analysis results of Harmony batch correction with prinicipal component analysis (PCA).
**Table S3.** Heterogeneity analysis results.
**Table S4.** Pleiotropy analysis results.

**Table S2.** Results of drug enrichment analysis for prognostic genes.

Data S3.

Data S2.

Data S1.

## Data Availability

The data used in this paper came from the GEO database (https://www.ncbi.nlm.nih.gov/geo/). The data that support the findings of this study are available from the corresponding author upon reasonable request.
