# Supplementary material for: Complementary multi‐omics profiling of chronic thromboembolic pulmonary hypertension reveals immune cell alterations, epigenetic changes, and genetically supported candidate genes
Source: Animal Model Exp Med. 2026 Mar 31;9(4):641–51. doi: 10.1002/ame2.70166 (PMC13242726; doi:10.1002/ame2.70166)
Supplement: Supplementary file 1 — Figure S1. The data collection and analysis process of this paper. Figure S2. Correlation analysis results of anthropometric characteristics and biochemical indexes. (A, B) anthropometric characteristics and biochemical index weight heat maps obtained by canonical correlation analysis (CCA) analysis. (C) The correlation analysis heatmap between the two. Figure S3. The basic analysis results of single‐cell RNA sequencing (scRNA‐seq) data. (A) The volcano plot obtained by screening highly variable genes using the Seurat software package. (B) The distribution of scRNA‐seq data from chronic thromboembolic pulmonary hypertension (CTEPH) and control group samples in a two‐dimensional nonlinear space after batch correction. (C, D) The distribution of cell clusters in a two‐dimensional nonlinear space after different types of cell labeling, based on the t‐SNE and UMAP dimensionality reduction methods, respectively. (E) The expression of the marker gene in different types of cells. (F) Bar chart showing the distribution of different cell clusters across various samples. Figure S4. The MR analysis results for differentially expressed genes (DEGs). Panels (A) through (G) show scatter plots of the MR analysis results for CLEC7A, COTL1, ETS1, FGR, LRP1, PILRA, and TNFSF13B with pulmonary arterial hypertension (PAH), respectively (the MR result for SAT1 was not plotted due to an insufficient number of SNP sites). (H) The MR analysis results for eight potential pathogenic genes. The horizontal dashed line corresponds to p = 3.59 × 10^−5^ (0.05/1394). “ln” stands for the natural logarithm; “OR” stands for odds ratio. “nSNP” represents the number of single‐nucleotide polymorphism (SNPs) used to estimate causal effects. “CI” stands for confidence interval. Table S1. Analysis results of Harmony batch correction with prinicipal component analysis (PCA). Table S3. Heterogeneity analysis results. Table S4. Pleiotropy analysis results. [file AME2-9-641-s004.docx]

**Supplementary Materials**

**1.1 Data Sources**

(1) Institutional data:

Clinical data were collected from 25 patients diagnosed with CTEPH at the Department of Cardiovascular Surgery, China-Japan Friendship Hospital. The dataset included anthropometric measurements (e.g., height, weight, BMI) and biochemical indicators (e.g., NT-proBNP, LDL, hemoglobin, troponin). Detailed clinical characteristics of these patients are summarized in Table (2) GEO database：

| **Data Type** | **GEO Accession** | **Sample Composition** |
| --- | --- | --- |
| **Single-cell RNA sequencing (scRNA-seq)** | **GSE274381** | 5 CTEPH samples and 2 healthy control samples |
| **Bulk RNA-seq** | **GSE130391** | 14 CTEPH samples and 4 healthy control samples |
| **DNA methylation array** | **GSE113061** | 5 CTEPH samples and 3 healthy control samples |

1. For the Mendelian randomization analysis, GWAS summary statistics were obtained from the FinnGen consortium (including 477 PAH cases and 372,077 controls), and eQTL summary data were retrieved from the IEU database.


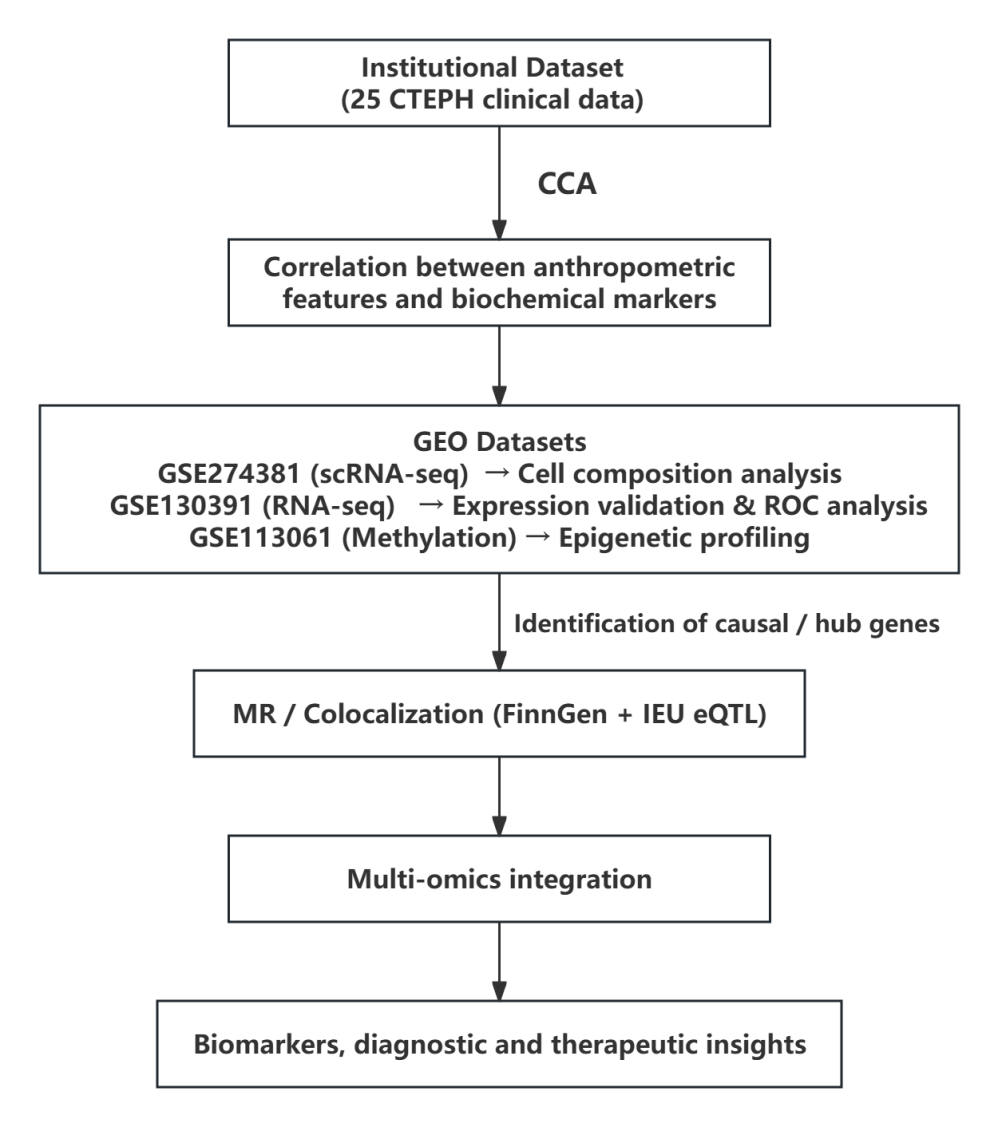


Figure S1 The data collection and analysis process of this paper


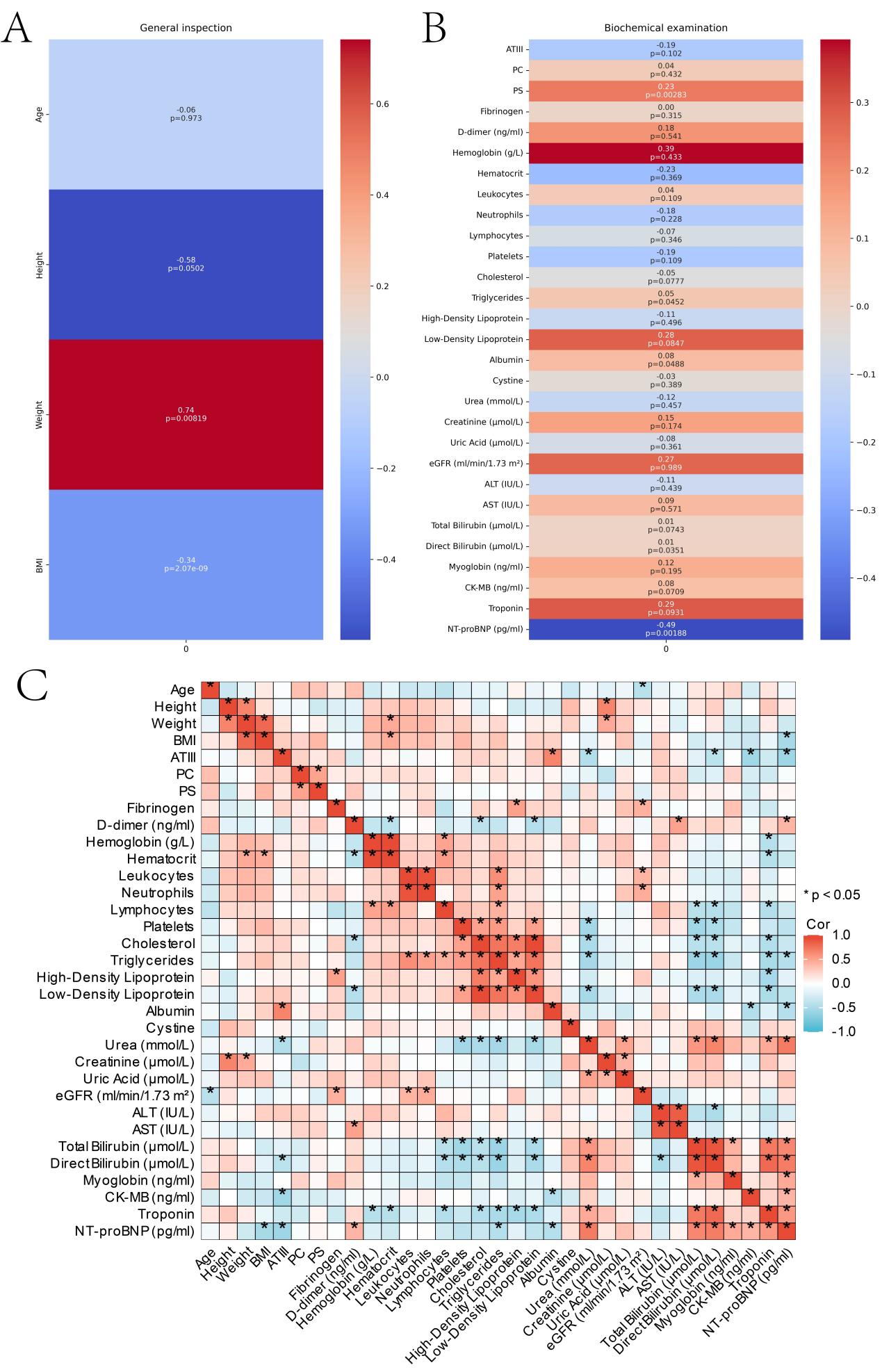


Figure S2 Correlation analysis results of anthropometric characteristics and biochemical indexes. A and B are respectively anthropometric characteristics and biochemical index weight heat maps obtained by CCA analysis. C is the correlation analysis heat map between the two.


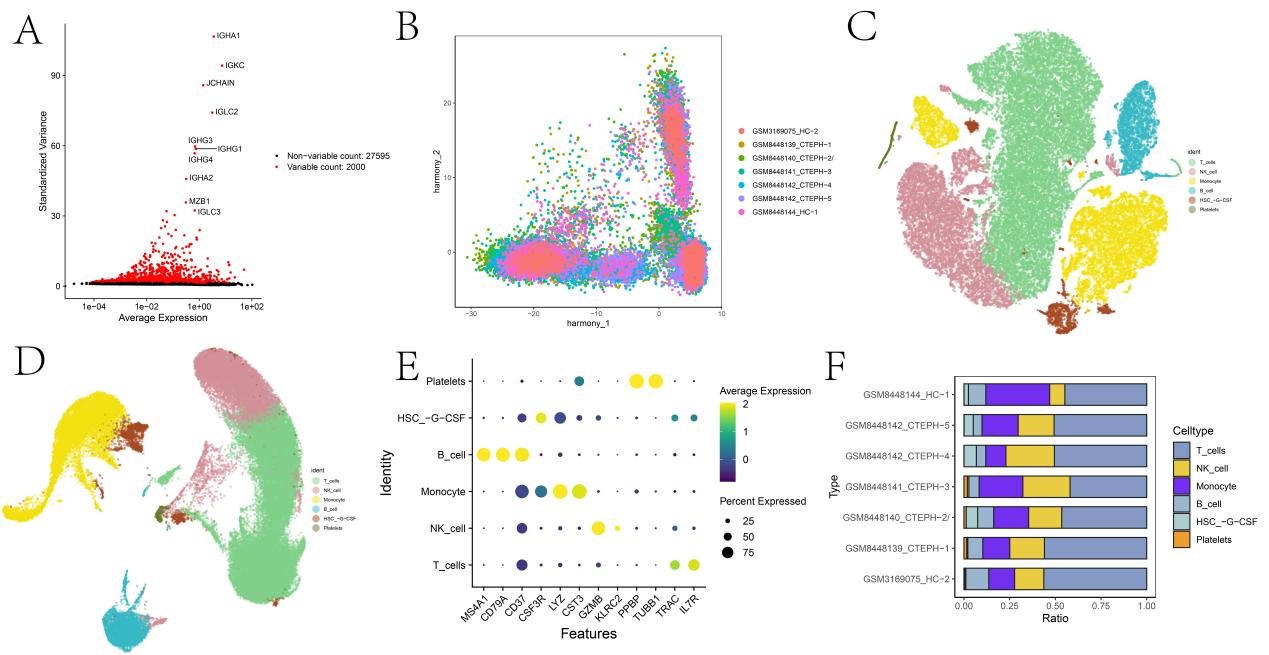


Figure S3 shows the basic analysis results of scRNA-seq data. (A) is a volcano plot obtained by screening highly variable genes using the Seurat software package. (B) illustrates the distribution of scRNA-seq data from CTEPH and control group samples in a two-dimensional nonlinear space after batch correction. (C) and (D) illustrate the distribution of cell clusters in a two-dimensional nonlinear space after different types of cell labeling, based on the t-SNE and UMAP dimensionality reduction methods respectively. (E) shows the expression of the marker gene in different types of cells. (F) is a bar chart showing the distribution of different cell clusters across various samples.


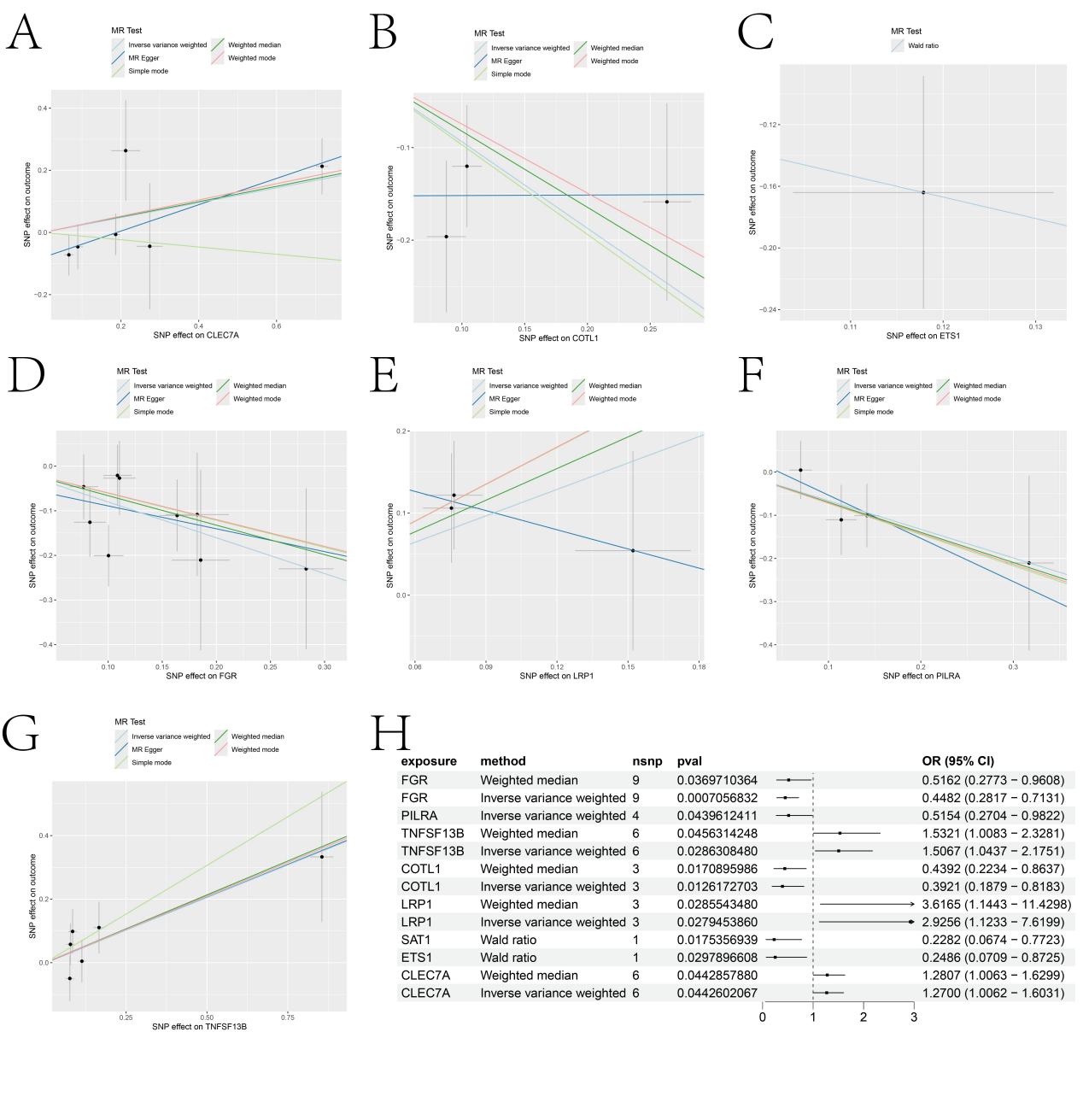


Figure S4 presents the MR analysis results for DEGs. Panels (A) through (G) show scatter plots of the MR analysis results for CLEC7A, COTL1, ETS1, FGR, LRP1, PILRA, and TNFSF13B with PAH, respectively (the MR result for SAT1 was not plotted due to an insufficient number of SNP sites). (H) summarizes the MR analysis results for eight potential pathogenic genes. The horizontal dashed line corresponds to P = 3.59 × 10^-5 (0.05/1394). 'ln' stands for the natural logarithm; 'OR' stands for odds ratio. 'nSNP' represents the number of SNPs used to estimate causal effects. 'CI' stands for confidence interval.

Table S1 Analysis results of Harmony batch correction with PCA

| Dimension | r2 | Reduction |
| --- | --- | --- |
| 1 | 0.0279950984677642 | PCA |
| 2 | 0.103296394346667 | PCA |
| 3 | 0.734713718857155 | PCA |
| 4 | 0.226272505808819 | PCA |
| 5 | 0.434774055329327 | PCA |
| 6 | 0.0766426197746503 | PCA |
| 7 | 0.175505731000874 | PCA |
| 8 | 0.148440570533111 | PCA |
| 9 | 0.0920121591064123 | PCA |
| 10 | 0.0585738763090962 | PCA |
| 11 | 0.015069630873965 | PCA |
| 12 | 0.031934118773253 | PCA |
| 13 | 0.0289539134142881 | PCA |
| 14 | 0.025835284778572 | PCA |
| 15 | 0.104388067065257 | PCA |
| 16 | 0.0397208166423011 | PCA |
| 17 | 0.0599869448717801 | PCA |
| 18 | 0.0158923008454036 | PCA |
| 19 | 0.0131220489816415 | PCA |
| 20 | 0.117179532723593 | PCA |
| 1 | 0.0211315477929447 | Harmony |
| 2 | 0.0125147116531812 | Harmony |
| 3 | 0.0144682909072223 | Harmony |
| 4 | 0.00802916404510579 | Harmony |
| 5 | 0.00637205365428839 | Harmony |
| 6 | 0.0182108612622556 | Harmony |
| 7 | 0.00133710116415087 | Harmony |
| 8 | 0.00767346000547964 | Harmony |
| 9 | 0.0068700357851218 | Harmony |
| 10 | 0.00178875984208648 | Harmony |
| 11 | 0.00217094108381999 | Harmony |
| 12 | 0.000779943508840364 | Harmony |
| 13 | 0.00491195905722419 | Harmony |
| 14 | 0.00151737779501993 | Harmony |
| 15 | 0.000476560367095757 | Harmony |
| 16 | 0.00122031908397668 | Harmony |
| 17 | 0.00132196561544828 | Harmony |
| 18 | 0.0021372184307008 | Harmony |
| 19 | 0.000300577868872609 | Harmony |
| 20 | 0.000935852406834914 | Harmony |

Table S3 Heterogeneity analysis results

| id.exposure | exposure | method | Q | Q_df | Q_pval |
| --- | --- | --- | --- | --- | --- |
| eqtl-a-ENSG00000000938 | FGR | MR Egger | 5.177 | 7 | 0.638 |
| eqtl-a-ENSG00000000938 | FGR | Inverse variance weighted | 5.375 | 8 | 0.716 |
| eqtl-a-ENSG00000085514 | PILRA | MR Egger | 0.552 | 2 | 0.758 |
| eqtl-a-ENSG00000085514 | PILRA | Inverse variance weighted | 0.770 | 3 | 0.856 |
| eqtl-a-ENSG00000102524 | TNFSF13B | MR Egger | 2.961 | 4 | 0.564 |
| eqtl-a-ENSG00000102524 | TNFSF13B | Inverse variance weighted | 2.962 | 5 | 0.705 |
| eqtl-a-ENSG00000103187 | COTL1 | MR Egger | 0.529 | 1 | 0.466 |
| eqtl-a-ENSG00000103187 | COTL1 | Inverse variance weighted | 2.744 | 2 | 0.253 |
| eqtl-a-ENSG00000123384 | LRP1 | MR Egger | 0.031 | 1 | 0.859 |
| eqtl-a-ENSG00000123384 | LRP1 | Inverse variance weighted | 1.310 | 2 | 0.519 |
| eqtl-a-ENSG00000172243 | CLEC7A | MR Egger | 2.799 | 4 | 0.591 |
| eqtl-a-ENSG00000172243 | CLEC7A | Inverse variance weighted | 5.177 | 5 | 0.394 |

Table S4 Pleiotropy analysis results

| id.exposure | exposure | egger_intercept | se | pval |
| --- | --- | --- | --- | --- |
| eqtl-a-ENSG00000000938 | FGR | -0.037 | 0.085 | 0.669 |
| eqtl-a-ENSG00000085514 | PILRA | 0.046 | 0.100 | 0.686 |
| eqtl-a-ENSG00000102524 | TNFSF13B | -0.0008 | 0.044 | 0.985 |
| eqtl-a-ENSG00000103187 | COTL1 | -0.152 | 0.102 | 0.376 |
| eqtl-a-ENSG00000123384 | LRP1 | 0.173 | 0.153 | 0.460 |
| eqtl-a-ENSG00000172243 | CLEC7A | -0.074 | 0.048 | 0.197 |
